# Stakeholder experiences and perspectives of genetic and genomic screening procedures in the Gulf Cooperation Council (GCC) region: a qualitative exploration

**DOI:** 10.1007/s12687-025-00819-x

**Published:** 2025-09-17

**Authors:** Safa Shaheen, Mohammed Ghaly

**Affiliations:** 1 College of Health and Life Sciences, Hamad bin Khalifa University, P.O. Box: 34110, Education City, Doha, Qatar; 2 Mohammed Ghaly Centre for Islamic Legislation and Ethics, College of Islamic Studies, Hamad bin Khalifa University, P.O. Box: 34110, Education City, Doha, Qatar

**Keywords:** Genetics, Genomics, Geneticization, Gulf Cooperation Council, Premarital screening, Whole genome sequencing

## Abstract

With swift progress in innovative fields like genetics and genomics, the application of genetic and genomic technologies in medicine has surged to unparallel heights, extending into domains traditionally beyond the scope of medical applications such as marriage. This phenomenon, known as geneticization, raises complex ethical, legal, and social implications. This study examines the social implications of geneticization in the Gulf Cooperation Council (GCC) countries, where premarital screening (PMS) is mandatory, and large-scale genome programs using Whole Genome Sequencing (WGS) have been underway for several years. Twenty-seven semi-structured, in-depth interviews were conducted with two stakeholder groups: Nine with professionals overseeing genetic and genomic initiatives and eighteen with participants of the PMS procedure. Key themes emerging from professional perspectives included child rights in genetic testing, awareness and knowledge gaps among public as well as professionals involved in counselling, stigmatization of the genetic disorders, and the future of genomics in the region. Insights from PMS participants highlighted the procedure’s impact on individual lives, concerns over privacy and confidentiality, the mandatory nature of the screening procedure, future reproductive plans for at-risk couples, and suggestions for the improvement of the PMS program. The findings offer valuable insights for improving the design and implementation of genetic and genomic screening programs in the region. Collaboration among policymakers, healthcare professionals, and the public is essential to address these challenges and enhance program effectiveness.

**Supplementary Information:**

The online version contains supplementary material available at 10.1007/s12687-025-00819-x.

## Introduction

The completion of the Human Genome Project (HGP) two decades ago stands as a major milestone in genetics and genomics. Sequencing the human genome through the HGP has significantly advanced genetic testing, drug design, gene therapy, pharmacogenomics, and more (Saleem 2020). The HGP was also commended for the emphasis on studying the ethical, legal and social implications (ELSI) arising from this monumental effort (Collins et al. 2003). This emphasis aimed to engage ethicists, social scientists, legal scholars, theologians, and other professionals in addressing emerging challenges related to advancing genome knowledge, ranging from societal and legal discrimination based on genetics to broader philosophical issues like genetic determinism (Collins and McKusick 2001).

Around the time of the HGP announcement in 1990, the concept of ‘geneticization’ was introduced by Abby Lippman, a Canadian epidemiologist and women’s health activist (Lippman 1991a). Her main concern was regarding the effects of adopting genetic technologies as part of routine health procedures especially prenatal genetic testing and its effect on women (Lippman 1993). Even though initially used to critique social anxieties related to genetic and biotechnological development, geneticization has evolved into a heuristic tool for individual, cultural, and social analysis. It influences notions of health, disease diagnosis, and prevention (Arribas-Ayllon 2016). Stemming from critiques of the growing use of genetic and genomic technologies, geneticization characterizes individuals based on their DNA sequences, giving rise to ethical, legal, and social challenges such as those related to human autonomy, equality, personal liberty, privacy, and accountability (Jensen et al. 2019). While ELSI studies have thrived in the Western context since the initiation of the HGP, there is a paucity of such studies in the Middle Eastern context. To comprehensively study the social aspects of technologies, it is crucial to involve different stakeholders, including the public, and examine their awareness, knowledge, acceptance, expectations, and attitudes toward the technology in question (Jensen et al. 2019).

WGS enables the identification of all the genetic variants present in the entire genetic content of a person. After the completion of HGP in 2013, many countries across the world have established national genome programs with results being returned to participants. Some examples are the United States of America, Estonia, Denmark and Japan (Stark et al. 2019). The integration of genomic sequencing into clinical settings is swiftly progressing, aided by significant government funding exceeding US$4 billion across a minimum of 14 nations (Stark et al. 2019). The national genome projects, which may vary sometimes in the usage of the procedures to sequence the genome, have been initiated with the goal of ultimately providing “personalized medicine” at the national level.

In the Gulf Cooperation Council (GCC) countries, where the incidence of genetic diseases is high, the discovery of all the disease-causing genes that affect the population in addition to understanding the gene-environment interactions promise to reduce the incidence of these diseases as well as provide tailored treatments to those affected. The use of the pioneering genomic tools to analyze the data generated from these highly consanguineous, inbred populations could generate new discoveries not seen in other populations around the world and could reshape the healthcare delivery in this region.

Against this backdrop, the various nationwide genome programs were initiated in the GCC countries, starting off in early 2010s as modest genome projects such as the Genome Arabia Project which aimed to sequence limited number of genomes from Qatar, Bahrain, Kuwait, United Arab Emirates, Tunisia, Lebanon, and Saudi Arabia. In the subsequent years, the nationwide genome projects were announced by Qatar, Saudi and Kuwait followed by UAE and Bahrain (Ghaly et al. 2016a).

On the other hand, premarital screening (PMS) refers to a set of examinations conducted on couples prior to marriage to detect genetic and infectious diseases, aiming to mitigate the risk of transmitting any such diseases to their offspring. PMS procedure has been implemented due to the high prevalence of autosomal recessive disorders such as hemoglobinopathies (Sickle Cell Disease, Thalassemia etc.) in the GCC region. These disorders account for a major proportion of physical and mental handicap (Teebi 2010) and are considered as significant burden on the healthcare systems due to their chronic nature that requires life-long medical attention, specialized care and expensive therapy (Al Kindi et al. 2012). Couples intending to marry are screened so that carriers of mutations causing these diseases can be identified. If both are carriers, despite being asymptomatic they can transmit the disease which can be expressed in the offspring. Transmission of sexually transmitted diseases can also be prevented via PMS. Couples with incompatible results undergo counselling so that they can make informed marriage choices including the consideration of not proceeding with the marriage plans.

In Cyprus, Greece, and Italy, premarital screening for thalassemia has been a longstanding practice due to high rates of consanguinity (Rahman et al. 2014). The successful implementation of the Thalassemia prevention program in Cyprus prompted other countries in the surrounding region facing similar issues to adopt the same model (Al-Arrayed et al. 1997). Mandatory PMS is implemented in the Islamic Republic of Iran, Malaysia, Jordan and Egypt, with varying conditions being screened for (Samavat and Modell 2004; Hamamy et al. 2006; Mitwally et al. 2000).

This article is part of a broader project investigating the ELSI of genetics and genomics in the GCC region. Numerous studies in Bahrain, Qatar, Kuwait, Oman, Saudi Arabia, and the United Arab Emirates (UAE) have examined key factors, including knowledge, awareness, and acceptance of procedures like PMS and WGS (Al-Shafai et al. 2022; Al-Farsi et al. 2014) (Al-Enezi and Mitra 2017).

Additionally, endeavors have been undertaken to assess the impact, outcomes, and effectiveness of nationwide genome projects and screening programs, including PMS (Almutawa and Alqamish 2009; Shaikha and Amani 2009); (Gosadi et al. 2021). In Qatar, we found a dearth of research on the outcomes of the compulsory PMS implemented since 2009 (Bener et al. 2019); (Alhosain 2018). Al-Shafai et al. supported this in their cross-sectional study on knowledge, perception, and attitudes toward the PMS program in Qatar (Al-Shafai et al. 2022).

## Materials and methods

### General approach and reasoning

This qualitative, semi-structured interview study used inductive, thematic analysis to understand stakeholder experiences and perspectives of genetic and genomic screening procedures in the GCC region. On identification of the gap of such studies in the region, a qualitative method was chosen to better characterize these aspects. This study was approved by the Qatar Biomedical Research Institute Institutional Review Board (QBRI-IRB-2023-3).

### Interview participants

We selected two distinct groups of interviewees: professionals specializing in genetics and genomics, and members of the public who underwent the genetic screening. The professionals, actively engaged in genetic and genomic programs, offer valuable insights into their experiences, lessons learned, and perspectives on the current status of these technologies in the GCC countries. Their expertise is crucial in assessing their comprehension of the geneticization phenomenon and their viewpoint on the extent to which society has embraced geneticization, along with its associated positive and negative consequences. On the other hand, members of the public, directly affected by these procedures, contribute unique experiences, expectations, and perspectives. Sharing their encounters provides a valuable grassroots perspective on genetic and genomic procedures, offering insights into personal impacts and concerns. This comprehensive approach enables us to capture a well-rounded view of the complex landscape of genetic and genomic practices within the GCC region.

### Measure development

The interview guides were developed based on a comprehensive literature review and analysis of the genetic and genomic screening procedures in the region. initially drafted by the first author who is a doctoral student. These were meticulously crafted based on insights from the literature review and were aligned with the primary research objectives. It was then reviewed and corrected by the second author who is a professor of biomedical ethics and religion and has been involved in individual research and collaborative projects regarding several different aspects of the genetic and genomic screening procedures in the region.

For the first set of stakeholders, interview questions covered their roles, institutional overviews, current and future initiatives, and evaluations of ethical, legal, and social aspects unique to each country. For the second set of stakeholders, the interview questions aimed to comprehensively explore participants’ experiences and perspectives of the PMS procedure. Additionally, we evaluated their satisfaction with existing programs, probed for concerns, including those related to privacy and confidentiality (please see attached interview guide as supplementary material).

### Procedures

For the first group of stakeholders, purposive sampling was employed to ensure the inclusion of professionals with relevant expertise and experience. The inclusion criteria required them to hold leadership positions within national genomic programs or premarital screening initiatives in a GCC country. These stakeholders were identified through professional networks and recommendations, primarily facilitated by the second author, who has extensive involvement in projects related to genetic and genomic programs in the region. Contact information, such as email addresses, was obtained from publicly available professional profiles. Potential participants were invited via email, which outlined the study’s aims and objectives. Upon their affirmative response, they were provided with a detailed informed consent form, which was duly signed before scheduling the interview. Interviews were conducted at times convenient for the participants.

For the second group of stakeholders, convenience sampling was utilized to facilitate participant recruitment. The inclusion criteria were that they had to be above 18 years of age and should have undergone the premarital screening procedure from one of the GCC countries and received the screening results. An email invitation was disseminated, encouraging interested individuals to participate in the interviews. The emails were sent out twice, with an interval of one month, utilizing both university communication networks (with which the researchers were affiliated) and personal networks to maximize outreach. The email included a link to a Microsoft form, where candidates provided basic information such as their name and contact email, if they were interested in participating in the study. Following submission of the form, participants received an informed consent document via email. They were invited to seek clarification if needed and, in the absence of queries, were requested to sign the consent form and propose a convenient time for the interview. As the list of potential participants primarily came from academic and personal networks, the sample may reflect individuals with higher educational backgrounds, better access to digital communication tools, and potentially greater health literacy.

The interviewer was contacted by each study participant on his/her initiative and all the interviews were conducted by the first author through the Zoom platform (Zoom Video Communications Inc.), scheduled at the convenience of the interviewees. The interviews were conducted between August and December 2022. Participants were repeatedly ensured that the interviews would be anonymized both during the recruitment phase and before recording the interview. To ensure deidentification, participants were requested to turn off the video and change their display name to ‘anonymous.‘. To ensure deidentification, professionals were identified as PRO_#, with # representing the numerical order of the interview, in subsequent sections when quoting their statements. Participants of PMS were labeled as ‘PAR_#’ where ‘#’ represented the interview’s numerical order. Only audio was recorded, transcribed, and saved securely on the primary researcher’s computer. Data was collected until saturation was achieved where no new codes or new themes emerged from the interviews.

### Analysis

Data analysis was guided by reflexive thematic analysis (Braun and Clarke 2006) and informed by the principles of Charmaz’s Grounded Theory (Charmaz 2006). This allowed for the exploration of the aspects of social phenomena that are of concern to the participants included in the study, (Engward and Davis 2015), specifically the concept of geneticization.

The analysis began after conducting a few initial interviews, which facilitated continuous refinement of the topic list, and the selection of participants based on preliminary findings. Memo writing was used to document emerging ideas throughout the analytical process. The analysis process was conducted in six phases:

In the first phase, the raw interview data was transcribed and formatted by the first author and thoroughly examined to comprehend emerging themes. The second phase involved generating initial codes based on the interview guide questions and identifying additional patterns by the first author. In the third phase, general categories were deduced from the interview questions, aligning with the research aims.

During the fourth phase, the first author performed inductive coding by which more specific categories were established. This was done through multiple readings of the raw data as well as by coding through NVivo (Version 12).

During phase five, the themes were defined and named after discussion and refinement among researchers, incorporating their subjectivity to enrich the interpretation. The first author who is a doctoral student of Indian origin and having spent most of her life in the GCC region, was well-positioned to understand the cultural nuances shaping participants’ lived experiences. Her unique position, as someone married outside the GCC and thus exempt from undergoing premarital screening (PMS), provided a perspective free from preconceived notions or bias regarding participant experiences. The second author, an Arab professor specializing in biomedical ethics and religion with extensive experience in individual and collaborative research on various aspects of genetic and genomic screening in the region, reviewed and refined the initial codes, further developing the themes and patterns. The thematic development involved an iterative process of comparison and re-examination of the interview data. This helped to pinpoint themes, categories, and identify interconnections for a comprehensive understanding.

Finally, in the last phase, the first draft of the report was created by the first author with input from the second author and the manuscript was reviewed and revised by both authors.

## Results

### Participant characteristics

Among the first set of stakeholders, a total of 14 professionals were identified and invited to participate, out of which 9 responded positively. 1 stakeholder from Oman was unable to participate due to scheduling conflicts during the interview period and subsequently referred a colleague in their place. 9 interviews were conducted, featuring two professionals each from Bahrain, Qatar, and Saudi Arabia, and one each from Kuwait, Oman, and UAE. The lower numbers in the latter three countries were due to a lack of response from invitees. All participants held senior positions in genetic testing services or were part of leadership teams for genomic projects.

The interviews ranged from 25 min to 1 h and 30 min each. All interviews, except for one, were recorded. One professional conveyed unease about the interview being recorded; consequently, key points were manually noted. The sociodemographic details are given in Table 1.

**Table 1:** Sociodemographic details of the interviewed professionals

Professional#	Sex	Country	PROFESSION
PRO_1	M	KUWAIT	Medical doctor
PRO_2	F	UAE	Human geneticist
PRO_3	M	SAUDI ARABIA	Human geneticist, medical doctor
PRO_4	M	SAUDI ARABIA	Member of Saudi Genome Program, genetic counsellor
PRO_5	M	QATAR	Human geneticist
PRO_6	M	OMAN	Member of National Bioethics Committee
PRO_7	M	QATAR	Human geneticist
PRO_8	F	BAHRAIN	Human geneticist, genetic counsellor
PRO_9	F	BAHRAIN	Human geneticist, family physician

In the second set of stakeholders, 20 participants of the PMS procedure initially responded to the invitation. However, 2 participants were excluded as their premarital screening was conducted outside the GCC region. Consequently, 18 interviews were conducted with eligible participants. Out of 18 participants,17 were female and 1 male, 13 were married and 5 were unmarried. Out of the unmarried, 4 were engaged with marriage plans in the near future and 1 had the engagement broken due to the incompatible screening result. The age range varied from 23 to 35 years and the educational level varied from high school to doctoral level. The interviews ranged from 15 to 45 min each. The demographic characteristics of this cohort are presented in Table 2.

**Table 2:** Sociodemographic details of interviewed participants (of PMS procedure)

Participant #	Sex	Age	Nationality	Educational level	Marital status
PAR_1	Female	31	Qatari	PhD	married, 2 children
PAR_2	Female	23	Yemeni	Bachelors	unmarried
PAR_3	Female	28	Jordanian	Masters	unmarried
PAR_4	Female	25	Iraqi	Masters	unmarried
PAR_5	Female	24	Yemeni	Bachelors	unmarried
PAR_6	Female	29	Qatari	Masters	married, 1 child
PAR_7	Female	28	Palestinian	Masters	unmarried
PAR_8	Female	27	Kenyan	PhD	married, 1 child
PAR_9	Female	29	Syrian	Masters	married, 1 child
PAR_10	Female	29	Jordanian	Masters	married
PAR_11	Female	27	Sudanese	Masters	married
PAR_12	Female	28	Indian	PhD	married
PAR_13	Female	35	Egyptian	PhD	married, 3 children
PAR_14	Female	35	Algerian	Masters	married, 1 child
PAR_15	Female	28	Jordanian	Masters	married, 1 child
PAR_16	Female	27	Jordanian	Masters	married
PAR_17	Female	28	American	Masters	married
PAR_18	Male	29	Nigerien	High School	married

### Interview themes

Based on the study’s objectives and the methodological assumptions utilized, a total of nine themes emerged from the interviews. Four themes were identified from interviews with the first set of stakeholders: (1) Child rights, (2) Awareness and knowledge gaps among the public and among counselling professionals, (3) Stigmatization, (4) Future of genomics in the region; and five themes were identified from interviews with the second set of stakeholders: (5) Impact of PMS on the lives of the participants, (6) Mandatory nature of the procedure, (7) Concerns about privacy and confidentiality, (8) Further plans if at-risk of begetting children with genetic disorders, and (9) Suggestions for improvement of the existing screening program. A notable thematic intersection emerged between Theme 3 and Theme 7, indicating that both stakeholder groups articulated concerns related to the social ramifications of genetic screening, particularly with regard to stigma, privacy, and confidentiality. All the identified themes are explained in further detail below:

Every interviewed professional emphasized the significance of integrating genomics and ethics. The consensus among the professionals is that advancing technology, particularly in genetic and genomic realms, without a robust ethical framework—or having a weak one—can have disastrous consequences. Among the most feared aspects of these technologies are eugenics and genome editing for non-medical and enhancement-related purposes:

There are some people [scientists] out there who believe that favorable characteristics should be selected- they justify [this] by saying that survival of the fittest is propagated by nature itself. Such people can misuse technology, and this is where the importance of ethical committees come in. (PRO_1, Kuwait)

Some of the main themes identified from the interviews with the professionals are mentioned below:

### Theme 1: Child rights

Given the distinct cultural and epidemiological characteristics of the region, there is a recognized need for regional regulations. Regarding the disclosure of carrier status information to children, international guidelines dissuade such practices due to minority status and the non-actionability of the information. Permission for such disclosure can only be granted by the individual upon reaching adulthood. In this region, parents express a desire to receive carrier status results that can be communicated to the child in a manner that is appropriate and acceptable.

Parents, they want to exercise their authority and of course their autonomy. But the child is still a minor. So the parents feel in this regard– ‘I think I should choose for my child because my decision is the best’. But it is not always like that. (PRO_8, Bahrain)

Hence, it is typically recommended for parents to refrain from conducting voluntary testing to determine the carrier status of their children. Additionally, in the event that this information is obtained, it is advised not to disclose it to the children to safeguard their autonomy as minors. Convincing Arab/Muslim parents of this guidance has proven challenging due to the strong sense of parental responsibility and the religious duty to ensure the child’s well-being, which encompasses a healthy upbringing. Consequently, parents often feel compelled to inform their children about their health status, believing it to be in the best interest of the child’s future. Another argument by parents, reports PRO_3, is that failure to disclose these results to parents can deprive the children from ever knowing these results if they are not available in the medical records. The availability of free/almost free healthcare also contributes to the increased practice of genetic testing.

Two genetic counselors interviewed expressed concern about prioritizing individual autonomy in marriage choices over the unborn child’s right to a healthy life, particularly in avoiding preventable conditions. A suggested solution is to educate prospective couples about the challenges of raising a diseased child, encompassing daily demands, psychological and financial strains on the family, etc. When considering the rights of the unborn child, there is a debate about acknowledging the fundamental human right to health. In certain situations, it was suggested that a balance must be struck between the right to autonomy in making marriage choices and the right to the health of the child.

And here you can face a lot of difficulties because in this part of the world a lot of people marry consanguineous since centuries. And for them, this is the custom. As a scientist explaining you know this is increasing risk for rare genetic disorders, I advise them that it’s better to consider to have a different partner. (PRO_8, Bahrain)

Premarital testing is not for the parents. It’s for the babies because the prospective parents are already considered healthy. They are only carriers. But this is better management of the baby’s health. The baby can get the cure once he’s born. So this can save his life. (PRO_4, Saudi Arabia)

### Theme 2: Awareness and knowledge gaps

One significant risk associated with advocating for genetic and genomic testing without adequate expertise is the potential for errors both in recommending the tests and interpreting results during counseling. The shortage of qualified and board-certified genetic counselors in the region exacerbates this issue. Some professionals in the field caution that relying on personnel trained in general medicine or other medical specialties to fulfill this role can be more hazardous than advantageous.

They indicate testing that is not on their, let’s say, responsibilities or duties or capacities just because they know about it…This is not an ethical practice that if you ignore science or if you don’t go deep into being absolutely sure what you say. So, if I do an erroneous counseling for a couple who is willing to marry, this is harming them.(PRO_8, Bahrain)

This underscores the importance of implementing effective awareness and training programs to enhance the competency of professionals in the field. Additionally, the appointment of certified genetic counselors is crucial to prevent errors and build public trust in these procedures.

Regarding public comprehension, there are two levels– awareness and knowledge. Awareness involves a vague familiarity with genetics, often acquired through routine procedures like compulsory PMS. The public generally doesn’t give much thought to these procedures. Media, such as movies, contribute to forming ideas about genetics, sometimes leading to misconceptions. In populations with high consanguinity and a prevalence of rare diseases, awareness alone is insufficient. There is a need to educate the public about the consequences of consanguinity, the likelihood of abortion, rare diseases, and the quality of life for individuals with these conditions.

### Theme 3: Stigmatization

Stigmatization occurs when individuals identified as carriers of a genetic disorder, along with their families, encounter social stigma and discrimination due to their carrier status. This becomes particularly significant when multiple family members are affected by a genetic disorder. Parents are anxious about potential societal ostracism and are also concerned about the marriage prospects of the unaffected children.

People start stigmatizing, you know and they said this family have this kind of disease, this family have the blindness, this family have any kind of genetic conditions. This was seen in remote areas- small villages…So stigma doesn’t come unless you are very close village and people know each other. People in villages have more stigma than the capital cities. (PRO_4, Saudi Arabia)

PRO_8, working in Bahrain also had the same view:

There is still a lot of stigma in this part of the world. People are so afraid that if someone else will find out that themselves or their children are affected by a disease, then they will be rejected from the society… Parents who are keeping children affected by rare disorders deprived at home, depriving them of the interventions that can bring a benefit and increase the quality of their lives because they are so much afraid that neighbors and other people will hear about this.

### Theme 4: Future of genomics

Genomic development in this region faced delays due to concerns about potential misuse of genomic information for creating ‘ethnic bioweapons’—weapons targeting specific ethnic groups. This concern originated in 1998 when a British newspaper, The Sunday Times, reported that Israel was allegedly developing an ethno-bomb to target Arab populations based on their genotype (Mahnaimi and Colvin 1998). Despite this report being fictional, it instilled initial hesitancy in Arab governments to adopt genomics on a large scale:

The ideology was that the genome is so importantly frightening. If you know the sequence, then you can make specific, targeted weapons against so and so, and so the governments around this area were all frightened. They lost the advantage basically because of this and it took a lot of education from the scientific community to convince them. (PRO_1, Kuwait)

Regarding the clinical utility of genomic results for complex diseases, it still has a long way to go, but we are not too far behind, says a senior geneticist and physician from Saudi Arabia,

You will find people very, very passionate on both sides of the conversation (regarding Polygenic Risk Scores). The good news is, you see, that professional societies like the American Heart Association is now inclined to incorporate the Polygenic Risk Score in the heart health and they did actually improve that. So that’s already a huge step forward. Also the biggest BRCA diagnostic laboratory for breast cancer-Myriad genetics- they now include the Polygenic Risk Score in their estimation of the breast cancer risk. That’s part of their clinical report. So there’s no question this is going to be the future. (PRO_3, Saudi Arabia)

From the interviewee responses, we were able to infer that a mixed approach is employed in the Middle East. Qatar and Saudi Arabia are using results based on PRSs to recommend lifestyle changes, and pharmacogenomics is being implemented in Qatar, Saudi Arabia, and Kuwait. Kuwait takes a more cautious approach in advising patients based on their genomic results, aiming not to create overly optimistic expectations regarding improvements in their current condition.

To build public trust, it’s crucial to deliver reliable genomic results. Staying updated on research, especially in multifactorial conditions supported by randomized clinical trials, is vital. Curating genotype-phenotype associations from literature ensures near accurate results and consistent advice, fostering trust in genomic testing outcomes. Recommendations include annual follow-ups, pretest and posttest consents for awareness, and caution with Direct to Consumer (DTC) testing companies, which may lack solid scientific backing. In the GCC region, DTC companies in this domain are limited.

The region has made significant strides in genetics and genomics, boasting cutting-edge infrastructure, global expertise, and local capacity building, placing it at the forefront of research and development. Collaborative efforts across interdisciplinary institutions have accelerated discoveries, particularly in treating rare genetic disorders. However, preventing such conditions remains challenging due to high consanguinity rates. Complex diseases like diabetes, coronary artery disease, and cancer still pose genomics challenges globally, and initiatives, including ethnicity-specific Polygenic Risk Scores (PRSs), are underway to address these complexities.

Personalized medicine is the future and in order to do the personalized medicine you need all the omics, and omics is basically genomics, proteomics,metabolomics, transcriptomics and microbiomics. I think within 10 years from now, doctors will prescribe medicine based on your genes. So I can see a very promising future in healthcare in terms of precise treatment and also reduction of the cost of every disease that we are managing right now. (PRO_2, UAE)

Precision medicine, personalized medicine, and predictive health all contribute to an increasing emphasis on geneticization. It is crucial to carefully prioritize which services should take precedence to ensure the optimal use of resources. An anticipated drawback is the potential for information overload, which could burden the healthcare system as individuals seek answers to questions raised by their genomic findings. The key challenge is to strike a balance.

Analyzing how the general population acquires knowledge about genetic and genomic processes within the country is essential to assess the extent of geneticization. Mandatory procedures, like PMS, gain widespread awareness due to legal requirements, with couples learning about it from relatives or friends. Short-term residents in GCC countries, unfamiliar with local marriage formalities, often rely on online sources or marriage officers at the ministry for information.

Some of the main themes identified after interviewing the participants of the PMS procedure are mentioned below:

### Theme 5: Impact on life

There were some participants who were heterozygous carriers for certain conditions like B-thalassemia major, such as PAR_7 and PAR_13. PAR_7 said that she became aware of her carrier status through premarital testing after which her marriage was cancelled, just like her brother’s marriage earlier. Having witnessed disease conditions in relatives, these participants prioritize preventing such conditions in their children, highlighting the significance of premarital testing in their lives.

We all find it (premarital screening) beneficial because it can give you an idea because we have in our family some affected members, they are major for beta thalassemia. And yeah, they did bone marrow transplantation and one of them died recently. So we understand the importance. (PAR_7)

The participants generally found this process impactful as it provided insight into their carrier status and genetic compatibility with their partners. However, a drawback is that it might mislead couples into thinking their children will not be affected by other genetic disorders which are not covered in the screening panel. Physicians or counselors should clarify the specific conditions screened for. The participants acknowledged that despite taking the form of a formality on the checklist for soon-to-be-married couples, this procedure is impactful in its own right.

### Theme 6: Mandatory nature of the procedure

Interviewed participants unanimously supported the mandatory status of the procedure, believing it encourages widespread participation. However, concerns were raised about the screening panel, with individuals from non-GCC ethnicities feeling that the screening is not tailored to their needs. A recurring issue was participants’ lack of awareness about the specific conditions being tested. Some participants desired this information but were not informed, indicating the routine nature of the procedure. Qatari citizens underwent testing in governmental clinics, while non-citizen residents were tested in private clinics. Some clinics offered pretest and posttest counseling, while others provided either of the two. In some cases, there was no formal counseling, and results were communicated through standard text messaging. Some participants desired additional tests and were willing to pay extra for them.

Some excerpts from the interviews regarding these aspects:

INTERVIEWER: Do you think this procedure (PMS) should be mandatory or optional?

PAR_3: It should be mandatory, but I suggest also if they can tell the people what the list of the conditions that they are screening for. Because like for me they didn’t mention anything. So, I think it’s good to inform the client or the person who is taking the test. I would like to know what conditions they are testing for and so we can suggest, for example, to include more conditions.

PAR_6: So I believe that if it’s intermarriages, it should be made mandatory, and then the option to get married or not will be optional, of course, but people need to be aware to make more studied decisions basically when getting married because they themselves may not have any symptoms or may not show the disease or the condition but then you find the children are suffering and are going for treatment.

PAR_11 had strong feelings when it came to marriage between at-risk couples:

I think it (PMS) should be mandatory, but I also think that it should be mandatory to ask people not to proceed with their marriage if there is a serious problem, especially if there’s a genetic problem. I think people who have high chances of producing disabled children or children with some genetic disease, they shouldn’t be allowed to proceed with their marriage.

PAR_17 expressed how the mandatory nature of the procedure is a blessing in disguise:

Since I did my bachelors in the public health field, there was always this notion, or I would say that we were advised to undergo PMS for STDs and also encourage our would be spouses to undergo the screening- but there was always this hesitation of asking the other party to do this, because this is not a comfortable suggestion to make especially before getting married, even though its very important. But since here it’s a mandatory procedure, that part got covered without having to bring up this topic for discussion.

The range of participant responses revealed differing views on the mandatory nature of the screening procedure. These ranged from full support to conditional endorsement in the case of consanguineous unions, to more stringent positions that discouraged marriage altogether when genetic incompatibility was identified.

### Theme 7: Concerns about privacy and confidentiality

Most of the participants were satisfied with the privacy and confidentiality aspects of the procedure and felt that both aspects were well managed by the clinic staff. Except three participants- PAR_5 who reported that the clinical staff offered to inform her of the fiancé’s test results. The reason for this is not known, but this does violate the confidentiality aspect of communicating the results and PAR_13 and PAR_12 who reported that the results were not shared separately but together. PAR_12 felt strongly about the confidentiality aspect of the PMS:

I feel there should be some level of confidentiality because what if this person decides not to go ahead with it (the marriage)? You know, and I mean at this point, like they’re not married or anything, so there is no requirement for them to disclose anything. (PAR_12)

Others, like PAR_16, who married a relative, believed that privacy and confidentiality were not a cause for concern since the results would be shared with the future spouse. This perspective, common in acquainted couples, contrasts with those arranged to marry strangers. This participant was informed about potential consequences of disclosing genetic status such as stigmatization. After this explanation, the participant acknowledged the importance of maintaining confidentiality until an individual chooses to share results with their future spouse:

Confidentiality is important, yes, but I think that the results should be given for them as a whole, like for the couple. I don’t see why it should be private, and each one has their own thing without the other one knowing. Because for example, let’s say in case that there is something positive in the test. So what’s going to happen next? My guess is they’re going to inform both parties that there is something wrong? (PAR_16)

INTERVIEWER: No, ideally the results will be revealed separately to each party and they will ask whether they want to do the counseling separately or together and they can choose to do it together or separately…Yeah, because sometimes there’s a problem of stigmatization. For example, if only the woman is affected, the man is not, and so and obviously you know the families are like close-knit. Sometimes it’s a tribal system and so the news would spread right? This girl has such a carrier status.

PAR_16: Yeah yeah, I see your point. I think you have a really good point. Especially if it’s kind of an arranged marriage. Yeah, I think, given this part of the world, given everything you said, yeah, I think it should be done separately.

### Theme 8: Further plans if at-risk

One of the questions that was asked to the participants was about the hypothetical scenario wherein if they would have proceeded with the marriage had the results been incompatible. Some participants, aware of options, expressed a preference for exploring alternatives like in vitro fertilization with preimplantation genetic diagnosis over ending the engagement if results were incompatible. When asked about the challenges—psychological, physical, and financial—associated with these procedures, the participants were unaware, but optimistic about overcoming these challenges with their partner’s support. Concerns about the rights of the child were raised by some participants, influenced by observations of friends, relatives, or work experiences:

I mean, yeah, if it was a situation like that, I would totally avoid it because then I know for a fact that this will happen. Yeah, and I have seen how difficult life can be. I mean if I have better choices then why do I have to make myself go through that, and it’s not just about me, it’s about the children’s lives as well, right? (PAR_3)

PAR_12 quoted the spiritual aspect of making the decision of whether or not to proceed with the marriage if both are found to be carriers:

Yeah, because I just feel like today I have the privilege of doing this testing and knowing what can happen in the future, right? But I also believe that with every disease, Allah has created a way to overcome it. So if we have the facility to know that this can happen in future for the kids, and then I would think that yes, there is a way probably around it. Maybe there already exists a treatment or something like that.

### Theme 9: Suggestions for improvement of existing screening program

Participants suggested improvements to the existing screening programs. Main concerns included a desire for screening for more genetic conditions, tailored testing for diverse ethnic backgrounds, and transparency about the screened conditions. Additionally, there was a perceived need for increased awareness about these disorders, particularly among younger girls, as they often marry early and become future caretakers for their children. This point was also raised by the professionals, but more than awareness, the process of really educating the public was suggested by them:

I think more awareness should be raised. Among young women, basically…because women have more considerations of children and if they think that something would threaten potential children then they might reconsider their options… (PAR_6)

I do think that to be more effective they should do more digging about a patient’s background. And tailor the testing to that person’s background. (PAR_8)

What made me a little bit shocked is that they only search for specific things here in Qatar.…. we have here in Qatar, lots of mutations and lots of consanguineous marriages so they can make it a little bit broader so people can understand.(PAR_9)

Other suggestions came in the form of including mental disorders and additional sexually transmitted diseases (STDs) in the screening:

I hope that they can just add couple of diseases, mental disorders because not everyone has genetic disorder. And also, I have come through a personal issue with STD and I heard many others suffering from STDs like HIV, Herpes since the day they were married. But they weren’t educated about it. (PAR_10)

I recommend having testing for the mental diseases and the psychological disorders. There should be a test to evaluate exactly if you have any trauma or if you have any kind of mental disorders because it will not be obvious… (PAR_15)

## Discussion

This study offers a nuanced understanding of the ELSI of genetic and genomic screening procedures in the GCC context, particularly focusing on the perspectives of both professionals and members of the public. Professionals leading these initiatives identified key challenges, including gaps in awareness and knowledge about the programs, concerns regarding child rights, potential stigmatization, and the future trajectory of genomics in the region. Simultaneously, program participants- those impacted by PMS- reflected on the program’s influence on their lives, sharing concerns about privacy and confidentiality, while also supporting the mandatory nature of the procedure. Participants also provided recommendations for enhancing the programs, such as incorporating more tailored testing options, including screening for additional sexually transmitted infections, mental health conditions, and other relevant disorders. These themes collectively point to a complex but evolving landscape shaped by local sociocultural values, religious beliefs, and biomedical advancements and resonate with earlier critiques that warn against overemphasizing genetic explanations at the expense of environmental, social, and behavioral determinants of health (Nelkin and Lindee^ 2010^; Sankar et al. 2004; Weiner et al. 2017).

The concept of geneticization, as first introduced by Lippman, warned of the increasing tendency to interpret health, behavior, and identity through a genetic lens (Lippman 1991b). This study finds that in the GCC region, geneticization is not merely a theoretical construct but a lived reality, especially through state-mandated programs like PMS. While professionals underscored the value of genetic screening for preventive health, there were notable concerns about its ethical implementation, especially regarding the balance between individual autonomy and collective health responsibilities.

The ethical tension between parental autonomy in marriage decisions and the rights of the unborn child was a prominent concern. The debate reflects ongoing global discussions about reproductive ethics in the genomic era, where autonomy must be weighed against public health interests and future generations’ welfare (Van der Hout et al., 2019). This issue becomes particularly complex in Islamic contexts, where parental responsibilities are deeply rooted in religious and cultural obligations (Ghaly et al. 2016b). Many couples proceed with marriages without understanding the potential consequences for their children, as was revealed by a survey conducted in Saudi Arabia where couples with affected children regretted their decision of proceeding with an at-risk marriage (Al Sulaiman et al. 2010). Participants’ views aligned with the idea that while individuals should have freedom in marital decisions, the potential suffering of offspring due to preventable genetic conditions raises an ethical consideration to act preemptively. However, considering genetic compatibility in spouse selection has also resulted in discrimination against individuals or family members with the same mutation. While mandatory screening promotes awareness, it may raise concerns about privacy and discrimination.

Encouraging responsible marriage choices and avoiding discrimination are ongoing challenges, along with ensuring at-risk couples heed advice from PMS results. When results indicate marriage risks, educating couples about the emotional, financial, and long-term challenges of having a child with a genetic disease is crucial. Tailored educational materials and interaction with families in similar situations can effectively emphasize the test’s importance and the need to prevent genetic diseases.

The issue of stigmatization, especially in tight-knit communities, remains a significant barrier to the full realization of genetic screening benefits. The effect of genetic labels is seen to be affecting family or tribe names as a genetic mutation or carrier status becomes attached to this name. This is consistent with previous research documenting how carrier status can lead to social exclusion, reduced marriage prospects, and secrecy (Rajab et al. 2007a). Additionally, parents from rural communities may feel a sense of inadequacy due to societal expectations and pressures related to family size and the burden of genetic disorders (Rajab et al. 2007b). This stigma is particularly strong in rural or conservative areas, where kinship ties and communal identity play a central role. Tackling this requires culturally sensitive strategies that normalize genetic screening as a proactive and responsible act rather than a marker of deficiency.

Participants expressed varying levels of concern regarding privacy and confidentiality. These concerns are well-founded, as any breach of genetic privacy could lead to instances of genetic discrimination or stigmatization. While most were satisfied with the protection of their data, specific breaches such as the shared disclosure of results or casual handling of sensitive information highlight areas needing regulatory oversight. These concerns are particularly salient in light of global debates about genetic privacy, especially as genomic data becomes increasingly digitized and integrated into national health systems (Bonomi et al., 2020). In the Western context, frameworks like the Genetic Information Nondiscrimination Act (GINA) in the U.S. offer some protections (Joly et al., 2020), but similar comprehensive legislation remains underdeveloped in many GCC states.

One of the clearest themes emerging from both professional and public stakeholders was the gap in genetic literacy. Professionals highlighted the inadequacy of training in genetic counseling, a gap that risks miscommunication and erroneous decision-making. This echoes prior findings emphasizing the need for comprehensive training and certification programs to ensure ethical practice in genetic medicine (Bensend et al., 2014). Public awareness was often shaped by limited exposure through state procedures or informal networks without a deeper understanding of genetic risk, screening limitations, or inheritance patterns. These gaps highlight the need for targeted public education programs, ideally integrated into school curricula or premarital counseling services (Holtkamp et al. 2017; Natarajan and Joseph 2021).

Stakeholders, particularly professionals, voiced optimism about the potential of personalized and precision medicine to transform healthcare. This aligns with global trends emphasizing pharmacogenomics, PRSs, and integrated omics approaches to better target disease prevention and treatment (Chen and Snyder 2013; Cross et al. 2022; Russell et al. 2021). However, the promise of such advances must be tempered with caution, especially concerning accessibility, clinical validity, and resource allocation. As stressed by the professionals interviewed, in regions with high rates of consanguinity, the utility of PRSs may be limited by the lack of representative population-specific data, necessitating more inclusive and diverse genomic research (Adam et al. 2023; Moreno-Grau et al. 2024).

### Study limitations and future research

The findings of this study should be interpreted within the context of several limitations inherent to qualitative research. The sample size, characteristics, and focus on a specific technology may limit the generalizability of the results to other populations. Additionally, the low male participation among the second group of stakeholders (participants of PMS) may reflect cultural factors in the region, such as limited male-female interaction, particularly with strangers. This reluctance to discuss personal aspects of their lives with unfamiliar individuals, particularly a female interviewer, aligns with observations by Harkness and Khaled in their study on societal norms surrounding consanguineous marriages, which highlight the private nature of citizens in the region (Harkness and Khaled 2014). Including a male interviewer to address gender-based barriers was considered; however, implementing this change would have required amendments to the consent form and Institutional Review Board (IRB) approval, which were not feasible within the study’s time constraints. Another limitation is the reliance on participant recruitment through the researchers’ affiliated university, which led to a sample population predominantly composed of highly educated individuals. This may not fully capture the diversity of experiences and perspectives within the broader population, particularly among individuals with varying levels of educational attainment. Future research should aim to address these limitations by recruiting participants from more diverse gender, educational, and socio-economic backgrounds. Such efforts would enhance the representativeness and generalizability of findings and provide a more comprehensive understanding of the phenomena under study.

## Conclusion

This qualitative study represents a preliminary effort in the region to explore the experiences and perspectives of stakeholders involved in various genetic and genomic screening programs such as PMS and WGS, and could meaningfully complement the existing quantitative research, offering richer insights into the area. The integration of genetic and genomic screening procedures in the GCC is not simply a medical or scientific matter but a deeply sociocultural and ethical one. This study underscores the necessity of grounding technological innovation within a framework that respects individual rights, cultural values, and community needs. As geneticization continues to shape the healthcare landscape, a participatory, transparent, and ethically robust approach will be essential to realizing the full potential of genetic screening and genomic medicine in the region. Policy development for these programs should incorporate the views of diverse stakeholders, especially those directly affected by the procedures. By fostering collaboration among genetics and genomics leaders, policymakers, religious scholars, ethicists, and social scientists, coupled with informed public engagement, it is possible to effectively navigate the ongoing implications of geneticization in the region.

## Electronic supplementary material

Below is the link to the electronic supplementary material.

Supplementary Material 1

## Data Availability

No datasets were generated or analysed during the current study.
