# Supplementary material for: Stakeholder experiences and perspectives of genetic and genomic screening procedures in the Gulf Cooperation Council (GCC) region: a qualitative exploration
Source: J Community Genet. 2025 Sep 17;16(6):813–25. doi: 10.1007/s12687-025-00819-x (PMC12569284; doi:10.1007/s12687-025-00819-x)
Supplement: Supplementary file 1 — Supplementary Material 1 [file 12687_2025_819_MOESM1_ESM.pdf]

SUPPLEMENTARY FILE

Journal of Community Genetics

Stakeholder experiences and perspectives of genetic and genomic screening procedures in the Gulf Cooperation Council (GCC) region: a qualitative exploration

Author information

1. Safa Shaheen (corresponding author)

   College of Health and Life Sciences, Hamad bin Khalifa University, P.O. Box: 34110, Education City, Doha, Qatar

   sshaheen@hbku.edu.qa

2. Mohammed Ghaly

   Centre for Islamic Legislation and Ethics, College of Islamic Studies, Hamad bin Khalifa University, P.O. Box: 34110, Education City, Doha, Qatar

   mghaly@hbku.edu.qa

INTERVIEW GUIDE

I. INTERVIEW WITH PROFESSIONALS

About yourself

• Would you please introduce yourself in few sentences?

• Since when are you affiliated with this program/organization, what is your role and main responsibilities in this regard?

About the organization

• Could you provide a short historical background of this program/organization, how it came into being and the factors that led to its establishment?

• How would you define the role of your program/organization in shaping the field of genomics in the region?

• How does your program/organization relate to other organizations/programs in the region? What makes one your program/organization unique or different from the other?

Present

• What are the main activities and initiatives run by your program/organization?

• Has the pandemic affected any of the activities or initiatives, and how?

Ethical, Legal and Social aspects

• How do you see the relationship between genomics and ethics?

• Do you think that the structure one's genome would have impact not only on one's health condition but also on other aspects, like one's character and behavior?

• What are the main ethical issues that you come across as part of working in the field of genomics and genetics?

• How would classify these ethical issues? Which issues can be seen as common or universal and which ones are peculiar to the socio-political and religio-ethical context of the region?

• As for the ethical management of incidental findings in particular, are there any existing regulations or policies in this regard?

• Are there any laws or national regulations that your organization have to abide by at the national or regional level?

• Have you ever experienced any cases where certain rights of research participants were compromised or violated? If yes, how were they addressed at the organizational level?

• How do you see the relation between the scientific ventures of your program/organization on one hand and the common values of the society on the other hand? Who is affecting who and how?

• What do you think about the concept of geneticization, and how its meaning would be different from cultural context to another?

Future

• What is the future direction of the field of genomics, globally, regionally and nationally?

• What there the main short- and long-term goals of your organization/program?

• Are there any other questions that you would like to address?

II. INTERVIEW WITH PARTICIPANTS (OF PMS)

• Could you give a little background information about yourself?

(Age, Nationality, Educational Background, Profession, Family details)

• How did you come to know about this procedure- PMS?

• Could you walk me through the procedure that you had to go through for PMS?

• What were the results? (compatible/not compatible)

• If positive, what would have been the decision had the results turned out negative? (Would you have still proceeded with marriage? Yes/No, Why?)

• Did this procedure have a considerable impact on your life? (Please elaborate)

• Is there any incidence of genetic diseases in the family (that have been seen in more than a generation)?

• Did any family members of older generation (ex. your mother) have to go through this procedure before their marriage?

- What is their opinion regarding this procedure?
- What is your opinion on whether this procedure should be mandatory?
- Did you have a concern about the privacy and confidentiality aspects of this procedure?
- Any other thoughts/points that you would like to share?
